# A function-blocking CD47 antibody suppresses stem cell and EGF signaling in triple-negative breast cancer

**DOI:** 10.18632/oncotarget.7100

**Published:** 2016-01-31

**Authors:** Sukhbir Kaur, Abdel G. Elkahloun, Satya P. Singh, Qing-Rong Chen, Daoud M. Meerzaman, Timothy Song, Nidhi Manu, Weiwei Wu, Poonam Mannan, Susan H. Garfield, David D. Roberts

**Affiliations:** ^1^ Laboratory of Pathology, Center for Cancer Research, National Cancer Institute, National Institutes of Health, Bethesda, MD, USA; ^2^ Cancer Genetics and Comparative Genomics Branch, National Human Genome Research Institute, National Institutes of Health, Bethesda, MD, USA; ^3^ Inflammation Biology Section, Laboratory of Molecular Immunology, National Institute of Allergy and Infectious Diseases, National Institutes of Health, Bethesda, MD, USA; ^4^ Center for Biomedical Informatics and Information Technology, National Cancer Institute, National Institutes of Health, Bethesda, MD, USA; ^5^ Laboratory of Cancer Biology and Genetics, Center for Cancer Research, National Cancer Institute, National Institutes of Health, Bethesda, MD, USA

**Keywords:** CD47, cancer stem cells, triple-negative breast cancer, epidermal growth factor receptor, therapeutic antibodies

## Abstract

CD47 is a signaling receptor for thrombospondin-1 and the counter-receptor for signal-regulatory protein-α (SIRPα). By inducing inhibitory SIRPα signaling, elevated CD47 expression by some cancers prevents macrophage phagocytosis. The anti-human CD47 antibody B6H12 inhibits tumor growth in several xenograft models, presumably by preventing SIRPα engagement. However, CD47 signaling in nontransformed and some malignant cells regulates self-renewal, suggesting that CD47 antibodies may therapeutically target cancer stem cells (CSCs). Treatment of MDA-MB-231 breast CSCs with B6H12 decreased proliferation and asymmetric cell division. Similar effects were observed in T47D CSCs but not in MCF7 breast carcinoma or MCF10A breast epithelial cells. Gene expression analysis in breast CSCs treated with B6H12 showed decreased expression of epidermal growth factor receptor (EGFR) and the stem cell transcription factor KLF4. EGFR and KLF4 mRNAs are known targets of microRNA-7, and B6H12 treatment correspondingly enhanced microRNA-7 expression in breast CSCs. B6H12 treatment also acutely inhibited EGF-induced EGFR tyrosine phosphorylation. Expression of B6H12-responsive genes correlated with CD47 mRNA expression in human breast cancers, suggesting that the CD47 signaling pathways identified in breast CSCs are functional *in vivo*. These data reveal a novel SIRPα-independent mechanism by which therapeutic CD47 antibodies could control tumor growth by autonomously forcing differentiation of CSC.

## INTRODUCTION

Breast progenitor cells play an active role in the cyclic changes that take place during pregnancy and ovulation in women [1, 2]. A minor subset of tumor cells has the capacity to initiate a new tumor upon transplantation into a healthy host. These tumor initiating cells have stem cell-like properties and are also known as cancer stem cells (CSCs). In contrast to CSCs, the bulk tumor cells have limited proliferative capacity and cannot form new tumors.

Despite advances in the diagnosis and treatment of breast cancer, these cancers frequently recur with a relapse time of 5-7 years [3]. One proposed mechanism is that CSCs are more resistant to chemoradiation therapies and persist in a dormant state during therapy but later initiate tumor regrowth. As few as 100 CD133^+^-expressing brain and breast cancer cells were sufficient to establish a new cancer in non-obese diabetic, severe combined immunodeficient (NOD-SCID) mice. In contrast, engrafted CD133^−^ cells did not form tumors [4, 5]. Flow cytometric analysis has shown that a CD44^high^ and CD24^low^ population is enriched in CSCs [6]. However, most existing therapies to treat solid tumors do not efficiently target cancer stem cells.

Breast cancers comprise four major molecular subtypes: luminal A, luminal B, triple negative/basal-like, and HER2 type [7]. Triple negative breast cancers (TNBC) represent approximately 20% of cases and are defined by their lack of expression of estrogen receptor (ER), progesterone receptor, and human epidermal growth factor receptor-2 (HER2). TNBC highly express epidermal growth factor receptor-1 (EGFR) and are highly proliferative, aggressive and resistant to systemic chemotherapies. Consequently, outcome for these patients is poor compared to ER^+^ and HER2^+^ cancers. Approximately 30-40% of deaths are caused by recurrence and metastasis of TNBC. Even though EGFR inhibitors have shown some promise for treating TNBC [8], no FDA approved targeted therapies have improved patient outcome for TNBC. Thus, there is urgency to identify signaling pathways required for TNBC and develop therapies targeting these pathways.

The ubiquitous cell surface protein CD47 is up-regulated in many cancers, and its high expression is a negative prognostic indicator for several cancers including invasive breast cancer [9, 10]. One proposed function of elevated CD47 expression on CSC is to serve as a “don't eat me” signal that protects the CSC from phagocytic clearance by macrophages [11]. Consequently, antibody and recombinant protein therapeutics that engage CD47 and block SIRPα binding have been developed to stimulate the destruction of CSC by macrophages [9]. The CD47 antibody B6H12 blocks the recognition of CD47 by its counter-receptor SIRPα on macrophages. Human tumors grown in immunodeficient NOD-SCID mice that express a variant of SIRPα that binds human CD47 with high affinity have been used to test the ability of B6H12 to enhance macrophage-mediated clearance of human tumor xenografts [10]. Inhibition of tumor growth by B6H12 in these models provided evidence to support the humanization of related CD47 antibodies for treating human cancer patients, which have now entered several human clinical trials (NCT02216409, NCT02367196, NCT02096770). Based in part on evidence that B6H12 has effects on CD47 signaling that are independent of blocking SIRPα binding [9, 12, 13], however, others have concluded that the CD47/SIRPα hypothesis is not sufficient to explain the antitumor activity of CD47 blockade and have reported that CD47 is more than a passive SIRPα counter-receptor.

Here we present evidence for an unanticipated activity of B6H12 in CSCs. We demonstrate a signaling role of the prototypical CD47 blocking antibody B6H12 on breast CSC but not differentiated cells isolated from the MDA-MB-231 cell line. These data provide evidence that this CD47 antibody can function independent of its known ligands thrombospondin-1 and SIRPα and should be considered a pharmacological agonist of CD47 signaling.

## RESULTS

### Characterization of breast CSCs derived from suspension cell-enriched MDA-MB-231 cells

**Figure 1:**
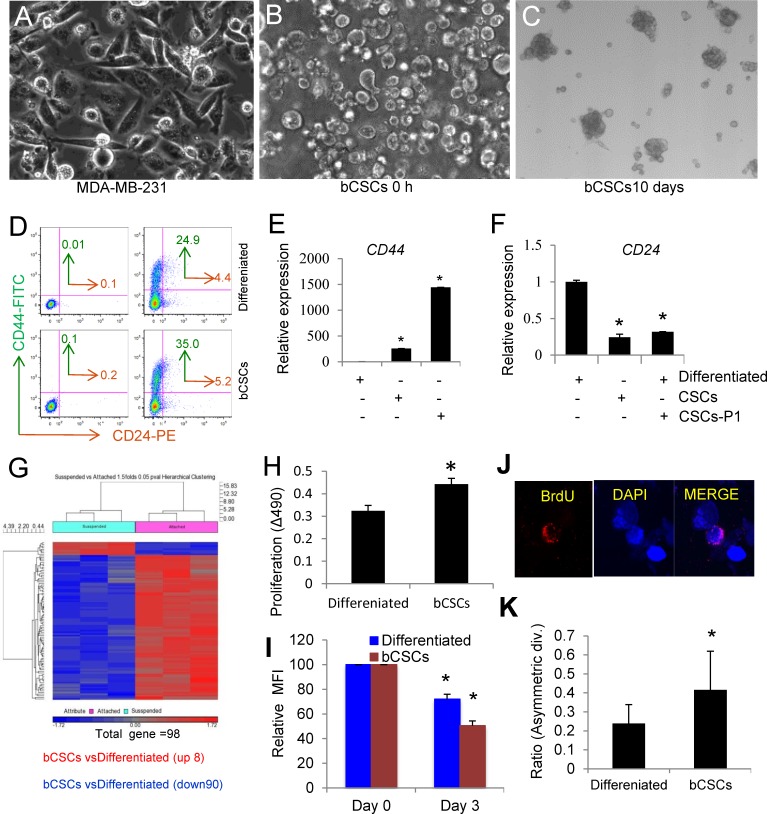
Characterization of breast cancer stem cells (bCSCs) derived from suspension cell-enriched MDA-MB-231 triple negative breast carcinoma cells **A**. Routinely cultured MDA-MB-231 cells showing loosely attached small round cells. **B**. With gentle agitation, loosely bound bCSCs were separated from adherent MDA-MB-231 cells. **C**. bCSCs form loose aggregates after incubation at 37°C for 10 days. **D**. Cell surface protein expression of CD44 and CD24 determined by flow. (**E**., **F**.) Replated bCSCs have higher CD44 and lower CD24 mRNA expression than control MDA-MB-231 cells. **G**. Hierarchical clustering of differentially expressed genes based on microarray analysis of MDA-MB-231 bCSCs versus unfractionated MDA-MB-231 cells. **H**. cell proliferation of differentiated cells and bCSCs were determined using a MTS assay. After 10 days bCSCs cells show significant increase in cell proliferation as compared to differentiated MDA-MB-231 cells (*p<0.05). **I**. Relative MFI of cell proliferation of differentiated cells (blue panel) and bCSCs (red panel) were analyzed using flow cytometry analysis from 0-3 days. Net MFI of differentiated MDA-MB-231 cells and bCSCs from 3 independent experiments were normalized to 100% at day 0 (*p<0.05). **J**. Representative image showing asymmetric division of BrdU-labeled (Red) MDA-MB-231 bCSCs after chasing with unlabeled BrdU and counterstaining with DAPI (Blue). **K**. Microscopic quantification of asymmetric cell division ratios for bCSCs and differentiated MDA-MB-231 cells (*p<0.05).

Routinely cultured MDA-MB-231 cells display abundant loosely attached round cells as well as firmly attached spread cells (Figure 1A). Previous studies have documented that the former cells form mammospheres, express characteristic bCSC markers, and are more tumorigenic in a murine xenograft model [14]. The loosely attached cells could be harvested by gentle shaking (Figure 1B) and formed aggregates within 10 days at 37°C in cancer stem cell medium (Figure 1C). The presence of CD44^high^ and CD24^low^ cancer stem/progenitor cells is a hallmark of aggressive metastatic TNBC [15]. As previously reported [14], the loosely attached cells expressed more surface CD44 than the firmly attached MDA-MB-231 cells (Figure 1D). Gene expression analysis by q-PCR of CD44 and CD24 mRNA in suspension and attached cells indicated that the suspension cells have 257-fold up-regulation of CD44 as compared to attached cells, and re-plating the suspension cells in stem cell medium further increased CD44 gene expression (Figure 1E). On the other hand, the suspension cells expressed 8-fold less CD24 than attached cells, which did not further change after re-plating (Figure 1F). Global microarray gene expression analysis of these two subsets indicated that loosely bound MDA-MB-231 cells differentially express many genes characteristic of CSC (Figure 1G). Among them, 8 transcripts were significantly upregulated (*P* = 0.05), and 90 transcripts were down regulated in suspension cells, including CD24. (Supplemental Table 1 and Supplemental Table 2). Based on these characteristics, we hereafter refer to the isolated suspension cells as bCSC and to the firmly attached cells as differentiated MDA-MB-231 cells.

We further performed a Gene Set Enrichment Analysis (GSEA) using existing stem cell gene signatures from the Broad Institute database. We then generated a list of stemness gene markers that were present at least in 3 different datasets and show an enrichment (either negative or positive) with the MDA-231 bCSC versus differentiated MDA-231 (Supplemental Table 3). The mRNA expression of some of these gene was then validated by q-PCR using differentiated and bCSCs cells from TNBC (Figure S1A-I). Consistent with previous reports of elevated CD47 in CSC [16-19] CD47 showed 2.3-fold higher expressions in bCSCs, whereas thrombospondin-1 and c-Myc, which is also suppressed in nontransformed cells by CD47 signaling [20], showed decreased expression in bCSCs (Figure S2A-S2C).

CSCs share some characteristics with embryonic stem cells. Correspondingly, real time PCR analysis of bCSCs revealed up-regulation of OCT4, Nanog, SOX2, and nestin relative to attached cells (Figure S2D-S2G). We further observed that bCSCs proliferate faster than differentiated MDA-MB-231 cells (Figure 1H and 1I), which is consistent with existing literature [14]. Another defining characteristic of stem cells is asymmetrical division. MDA-MB-231-derived CSCs divide asymmetrically for self-renewal [21], and asymmetric division is correlated with the CD44^high^/CD24^low^ phenotype [22]. We chased BrdU-labeled bCSCs with unlabeled BrdU to quantify asymmetric DNA template strand segregation [23]. Differentiated MDA-MB-231 cells and bCSCs were labeled with BrdU for two weeks and chased for 2 divisions in BrdU-free medium. The cells were treated with cytochalasin D, and symmetric versus asymmetric DNA segregation was counted microscopically. bCSCs enriched for CD44^high^CD24^low^ showed an increase in asymmetric cell division (Figure 1J-1K).

### CD47 antibody B6H12 inhibits bCSC proliferation, asymmetric division, and expression of KLF4

**Figure 2:**
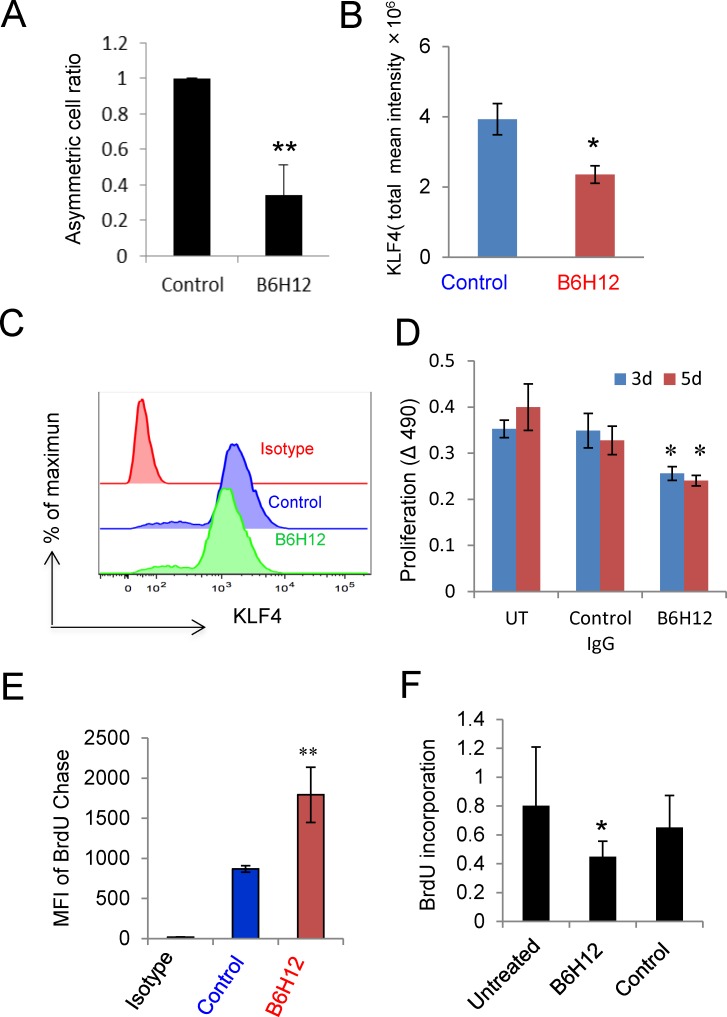
B6H12-Ab inhibits asymmetric cell division and cell proliferation **A**. B6H12-Ab and control antibody treated cells were immunostained using BrdU antibody, and the asymmetric cell division ratio was scored microscopically (*p<0.05). **B**. MDA-MB-231 and bCSCs cells were treated with B6H12 or isotype control antibody for 24 h and immunostained using KLF4 antibody. Total mean intensity of KLF4 positive cells was calculated, and t-test was performed. **C**. Flow cytometry analysis of KLF4. (D) Cell proliferation of bCSCs were determined using MTS assay for 3 and 5 days. B6H12 Ab treated bCSCs cells show significant decrease in cell proliferation as compared to untreated and control (*p<0.05). **D**. Quantification of BrdU staining by flow cytometry. **E**. CD44^high^/CD24^low^ sorted bCSCs derived from MDA-MB-231 were labeled with BrdU for 24 h and further treated with B6H12 (1 μg/ml) for 24 h. BrdU incorporation was measured using a BrdU cell proliferation assay kit.

To observe the effect of B6H12 on asymmetric cell division, bCSCs were labeled with BrdU and chased using BrdU-free medium in the presence of B6H12 or control antibody. The cells were immunostained using anti-BrdU and quantified using confocal microscopy imaging (Figure 2A). The fraction of cells exhibiting asymmetric division significantly decreased after B6H12 treatment.

We further analyzed effects of B6H12 on expression of the embryonic stem cell markers OCT4, SOX2, NANOG and KLF4 in differentiated cells and bCSCs. OCT4, SOX2, and NANOG immunostaining did not change between isotype control and B6H12 treatments as well as in microarray analysis (data not shown), KLF4 decreased moderately in differentiated cells (Figure S3A and S3C), but a statistically significant reduction of KLF4 was observed in bCSCs (Figure 2B and Figure S3B). Decreased KLF4 protein expression was confirmed using flow cytometry (Figure 2C). Because KLF4 is an essential gene for breast cancer stem cell maintenance and it's decrease leads to decreased proportion of stem/progenitor cells [24], B6H12 treatment may reduce the number of bCSC by down-regulation of KLF4, leading to the observed inhibition of asymmetric cell division.

To further investigate direct effects of the CD47 blocking antibody B6H12 on bCSCs, we cultured differentiated MDA-MB-231 cells and bCSCs in the presence of B6H12 or an isotype-matched control IgG for 3 days. The CD47 antibody decreased the number of round non-adherent bCSCs cells (arrows) but had no effect on the morphology of differentiated MDA-MB-231 cells (Figure S4A-S4B). B6H12 also reduced the size of mammospheres when bCSCs were cultured for 10 days using cancer stem cell medium (Figure S4C-S4E).

**Figure 3:**
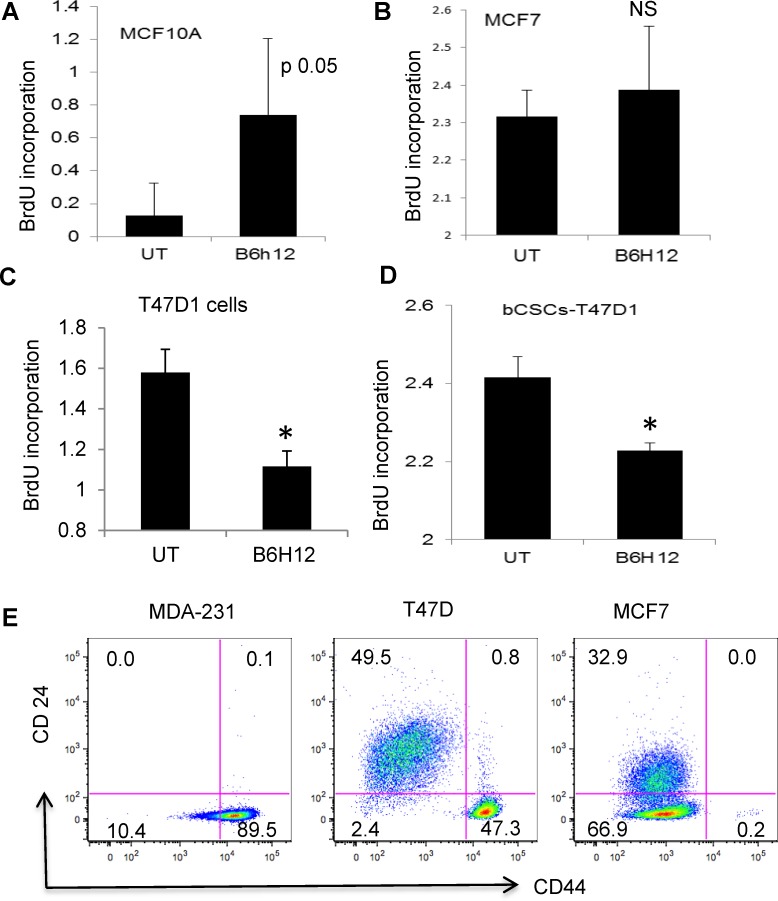
B6H12 specifically targets breast carcinoma cells that have high number of bCSCs/tumor initiating cells The following cell lines were treated with 1 μg.ml B6H12, and DNA synthesis was quantified by BrdU incorporation: **A**. MCF10A **B**. MCF7 **C**. T47D1 and **D**. bCSCs sorted for CD44^high^ CD24^low^ sorted bCSCS derived from T47D1 (C) The cells were labeled with BrdU for 24 h. BrdU treated cells were further treated with B6H12 Ab (1μg/ml) for 24 h. The BrdU incorporation was measured using a BrdU cell proliferation assay kit. **E**. MDA-MB-231 cells have a higher percentage of CD44high/CD24low cells than MCF7 and T47D1cells. T-test P value ≤ 0.05(*). NS(non-significant).

Proliferaton of the bCSC after 3 or 5 days was inhibited in the presence of B6H12 but not the isotype control antibody (Figure 2D). Flow cytometric analysis of equilibrium BrdU-labeled bCSCs chased with unlabeled BrdU showed that B6H12 treatment significantly limited the mean dilution of BrdU compared to isotype control antibody (Figure 2E). This confirms that ligation of CD47 by B6H12 initiates an anti-proliferative signal in bCSC. We examined the specificity of the antiproliferative activity of B6H12 for TNBC by testing two ER^+^ breast cancer cell lines (MCF7 and T47D) and a normal immortalized breast epithelial cell line (MCF10A). Consistent with our published studies using lung endothelial and T cells, the CD47 blocking antibody B6H12 increased DNA synthesis in MCF10A cells (Figure 3B). MCF7, a well-differentiated ER^+^ breast carcinoma cell line with limited malignant potential, also exhibited a positive response to B6H12 (Figure 3A). However, the breast carcinoma cell line T47D and sorted T47D-bCSC showed a similar inhibition of proliferation by B6H12 as MDA-MB-231 bCSC (Figure 3C-3D). To verify the sensitivity of bCSC derived from MDA-MB-231 and T47D, we isolated CD44^High^/CD24^low^ cells by sorting and assessed BrdU incorporation. B6H12 significantly inhibited cell proliferation of these purified bCSCs derived from MDA-MB-231 and T47D cells (Figures 2F and 3D). This data indicates that B6H12 specifically targets CD44^high^ and CD24^low^ subsets of cells in triple negative and certain ER^+^ breast cancers that have more tumor initiating cells but is not effective on other ER positive breast cancers or normal breast epithelial cells (Figure 3E).

**Figure 4:**
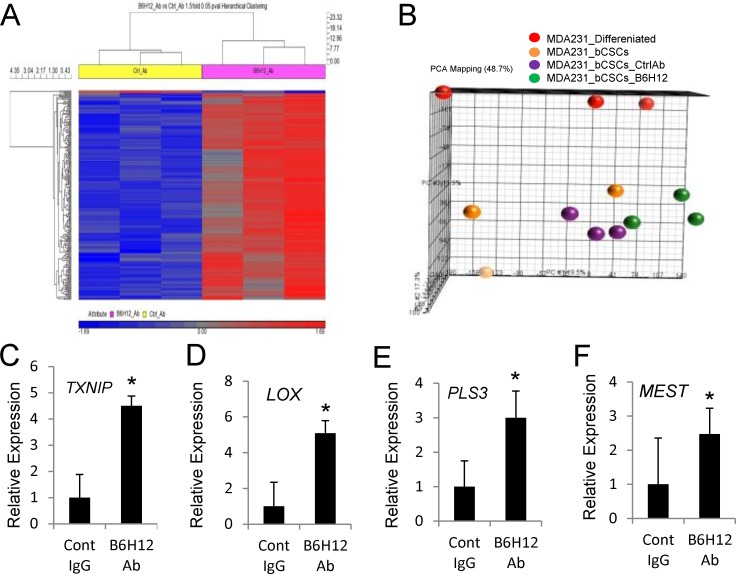
B6H12 alters gene expression of bCSCs **A**. Hierarchical clustering of microarray data comparing bCSCs treated with control IgG or B6H12 for 36 h. **B**. Principal component analysis of MDA-MB-231 bCSCs, differentiated MDA-MB-231 cells, bCSCs treated with anti-CD47 B6H12 and control IgG. (**C**.-**E**.) Real time PCR validation of GSEA enriched expressed genes between bCSCs vs differentiated MDA_MB-231 cells analyzed in bCSCs treated with control IgG or B6H12 for 3 days

Global microarray assessment of mRNA expression revealed that B6H12 significantly altered the expression of 225 transcripts as compared to isotype control antibody (Figure 4A). Principle component analysis showed that B6H12 antibody induced a distinct gene expression signature in bCSC relative to control antibody-treated and differentiated MDA-MB-231 cells (Figure 4B). Several of the genes that showed altered expression in bCSC versus differentiated MDA-MB-231 cells (Figure S1A-S1I) were reversed following B6H12 treatment (Figure 4C-4F). B6H12-treated bCSCs showed an up-regulation of thioredoxin-interacting protein (TXNIP, Figure 3C) and LOX (lysyl oxidase, Figure 4D), which are known tumor suppressors [25, 26]. Altered TXNIP mRNA expression was previously reported following CD47 knockdown in hepatocellular carcinoma stem cells [17]. Similarly, B6H12 treatment selectively increased bCSC expression of plastin-3 (PLS3, Figure 4E), which is associated with actin and calcium ion binding [27, 28]. Loss of mesodermal-specific transcript MEST/PEG1 has been associated with invasive breast cancer [29]. MEST/PEG1 showed down regulation in bCSCs (Figure S1I), and B6H12 treatment significantly increased expression 2.5-fold over control antibody treatment (Figure 4F).

### B6H12 down-regulates components of the EGFR pathway in bCSCs

**Figure 5:**
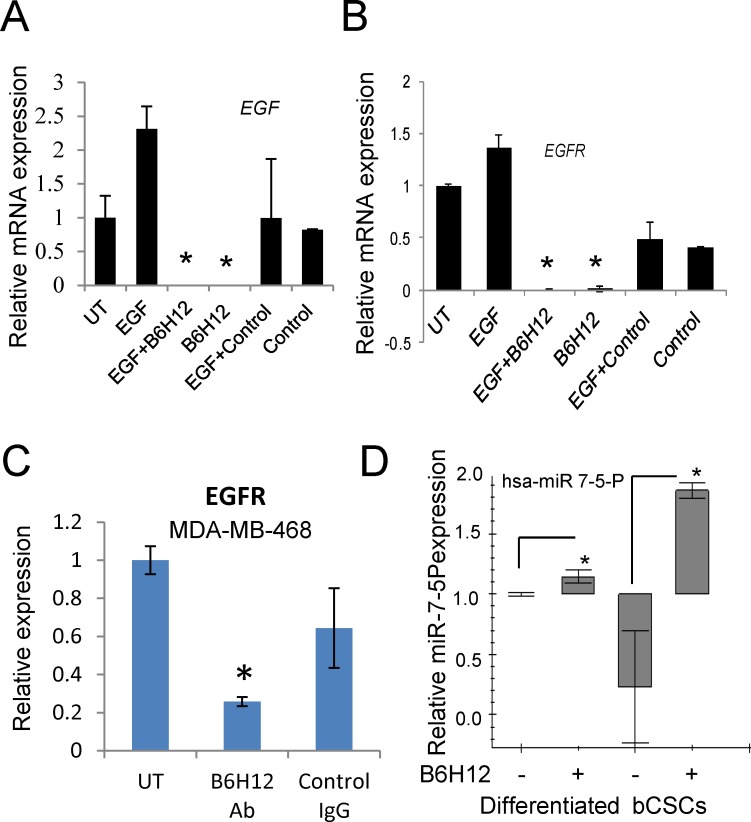
B6H12 suppresses EGF and EGFR mRNA expression in bCSCs EGF **A**. and EGFR mRNA expression **B**. is up-regulated in bCSCs, and down-regulated by B6H12 antibody treatment. EGF and EGFR mRNA expressions were undetectable in bCSCs treated with B6H12 Ab. **C**. EGFR mRNA expression in MDA-MB-468 cells treated with B6H12 or isotype control IgG. **D**. Differentiated MDA-MB-231 cells and bCSCs cells were treated with B6H12 Ab for 24 h. Total miRNA was extracted, and miR-7 was analyzed using real time PCR. Relative expression of hsa-miR 7-5P was measured from differentiated and bCSCs, and SNO47 was used as control for normalization.

High expression of EGFR is characteristic of ER^−^ breast tumors and has been linked to poor prognosis [30-32]. ER^−^/HER2^low^ cancers with a EGFR^high^ phenotype were reported to have a higher number of stem/progenitor cells [30, 33, 34]. Treatment of bCSCs with B6H12 for 3 days down-regulated EGFR at the mRNA level on microarray analysis (Supplemental Table 4). Real time PCR confirmed that treatment with B6H12 alone or in the presence of EGF eliminated detectable EGF and EGFR transcript expression (Figure 5A, 5B), whereas treatment with isotype control antibody in the presence or absence of EGF did not significantly alter mRNA expression of EGF and EGFR. Specific suppression of EGFR mRNA expression by B6H12 was reproduced in a second TNBC cell line (MDA-MB-468, Figure 5C).

The microarray data indicated that several additional genes involved in signaling or regulation of the EGFR pathway including NFAT5, CCDC88A/girdin, API5, EEA1, and IQGAP1 were significantly induced in the bCSC by B6H12 treatment (Supplemental Table 5), but these could not directly account for the strong down-regulation of EGFR mRNA. However, we noted that both EGFR and KLF4 mRNAs are known targets of microRNA-7 (miR-7) in human cancer cells [14, 35]. IGF1R mRNA is another known target of miR-7 and was also significantly altered by B6H12 (Supplemental Table 5). This suggested a potential mechanism by which B6H12 decreases EGFR expression in TNBC. To investigate if B6H12 treatment alters expression of miRNA-7, we measured expression of has-miR-7-5P from differentiated MDA-MB-231 cells and bCSCs cells. B6H12 significantly increased miR-7-5P expression in bCSC but had minimal effects on differentiated MDA-MB-231 cells (Figure 5D). Dicer was also upregulated in B6H12 treated bCSCs (Supplemental Table 5). Dicer is a component of a complex that cleaves dsRNA precursors to yield functional miRNAs (21-23). bCSCs express low levels of RNA-induced silencing complex (RISC) genes as compared to differentiated cells (Figure S5A-S5D). However, B6H12 increased mRNA expression of Ago2, DICER, DGCR8 and Drosha but not Ago1 (Figure S5E).

### B6H12 acutely regulates EGFR signaling

**Figure 6:**
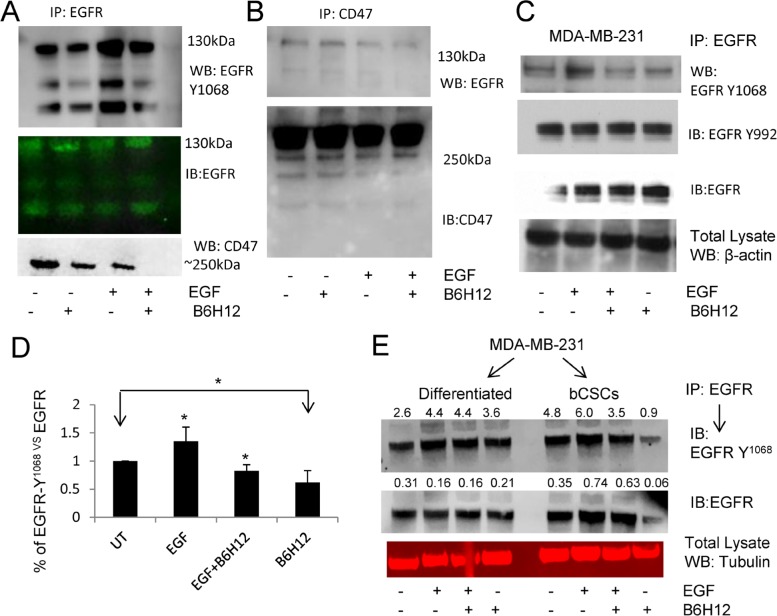
**A.** AEGFR-immunoprecipitation from MDA-MB-231 cell extracts followed by western blotting shows that B6H12 treatment for 15 min disrupts the association between EGFR and CD47 and inhibits EGFR-Y^1068^ phosphorylation. **B**. CD47-immunoprecipitation showed that a fraction of EGFR co-immunoprecipitates with EGFR. B6H12 treatment for 15 min reduced interaction between CD47 and EGFR in MDA-MB-231 cells. (**C**.-**D**.) MDA-MB-231 cells were pretreated with B6H12 for 15 minutes followed by EGF for 5 minutes, and IP-western blotting was performed using phospho-EGFR antibody. **D**. Quantification of three experiments was analyzed using the t-test (*P<0.05). **E**. Extracts from differentiated MDA-MB-231 cells and bCSCs treated as indicated were subjected to EGFR immunoprecipitation as in panel A. One representative blot of 3 independent experiments is shown. Numbers above lanes indicate quantiation by densitometry.

Based on our previous finding that CD47 laterally associates with the tyrosine kinase receptor VEGFR2 [36], we asked whether CD47 similarly interacts with EGFR. Immunoprecipitation revealed that EGFR and a small fraction of CD47 co-immunoprecipitate, and pretreatment with B6H12 antibody disrupted this interaction and inhibited EGFR-Y^1068^ phosphorylation (Figure 6A-6B). To determine whether B6H12 treatment acutely altered EGFR tyrosine phosphorylation, we pre-treated MDA-MB-231 cells for 15 min with B6H12. EGF stimulation for 5 min significantly increased EGFR phosphorylation at Y^1068^. B6H12 treatment in the presence or absence of EGF inhibited EGFR phosphorylation at Y1068 but not Y^992^. B6H12 treatment alone inhibited basal EGFR Y^1068^ phosphorylation as compared to untreated (Figure 6C-6D). Similarly, differentiated cells and bCSCs derived from MDA-MB-231 cells were treated with either EGF alone or in combination with B6H12. B6H12 treatment inhibited basal and EGF-stimulated EGFR phosphorylation in bCSCs but not in differentiated cells (Figure 6E). We also examined Y^998^ phosphorylation but did not observe any change (Figure S6A), which is consistent with Figure 6C. To further validate these results, we isolated a pure population of CD44^high^/CD24^low^ MDA-MB-231 cells by cell sorting. Stimulation of FACS-sorted bCSCs with EGF did not further increase EGFR Y^1068^ phosphorylation, but B6H12 strongly inhibited EGFR Y^1068^ phosphorylation in the absence and presence of co-stimulation with EGF (Figure S6B). Significant inhibition of EGFR Y^1068^ phosphorylation was observed using either tubulin or total EGFR for normalization (Figure S6C-S6D). These data demonstrate that B6H12 acutely targets EGFR signaling in bCSCs by down-regulating EGFR phosphorylation.

### Stem cell and cell death markers correlate with CD47 expression in human breast cancers

**Figure 7:**
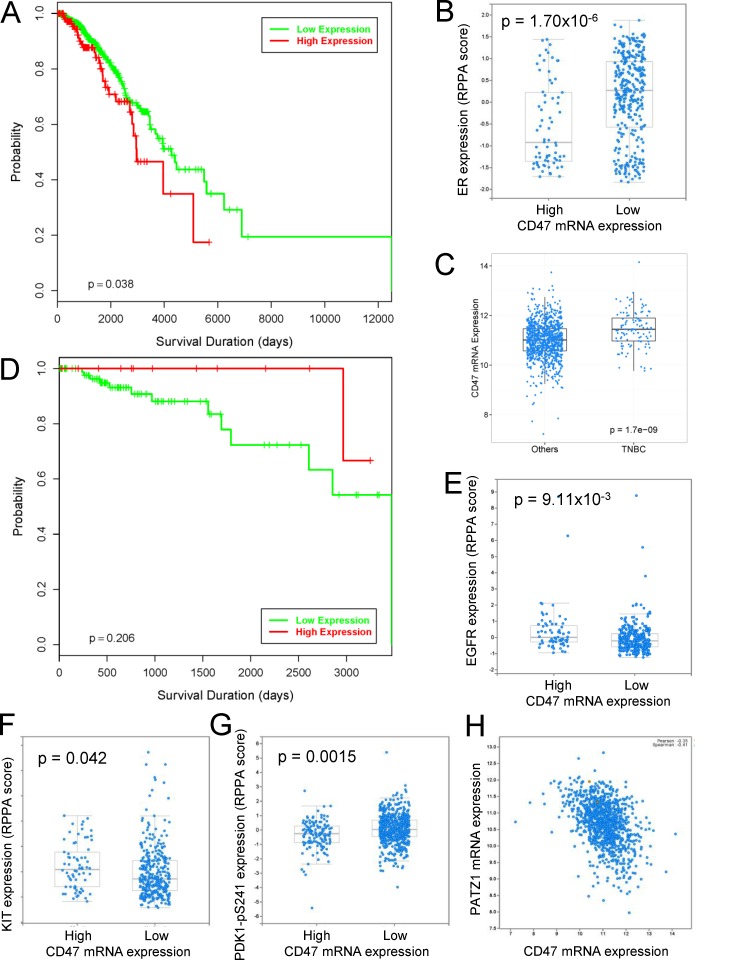
Analysis of CD47 expression in the TCGA invasive breast cancer data **A**. Kaplan–Meier survival curves for breast invasive carcinoma patients comparing those with CD47 mRNA expression determined by RNAseq >1 SD higher than the mean (red, n=152) and those without increased CD47 expression (green, n=943). **B**. TCGA expression data for estrogen receptor in breast tumors determined by reverse phase protein arrays (RPPA) stratified by CD47 mRNA expression. **C**. Comparison of CD47 mRNA expression in TNBC (n=113) versus other subtypes (n=982). **D**. Kaplan–Meier survival curves for TNBC patients comparing those with CD47 mRNA expression determined by RNAseq >1 SD higher than the mean (red, n=15) and those without increased CD47 expression (green, n=98). **E**.-**H**. TCGA expression data for the indicated proteins in breast tumors determined by reverse phase protein arrays (RPPA) stratified by CD47 mRNA expression. P-values in panels B, E-G were based on 2-sided 2 sample t-tests.

**Figure 8:**
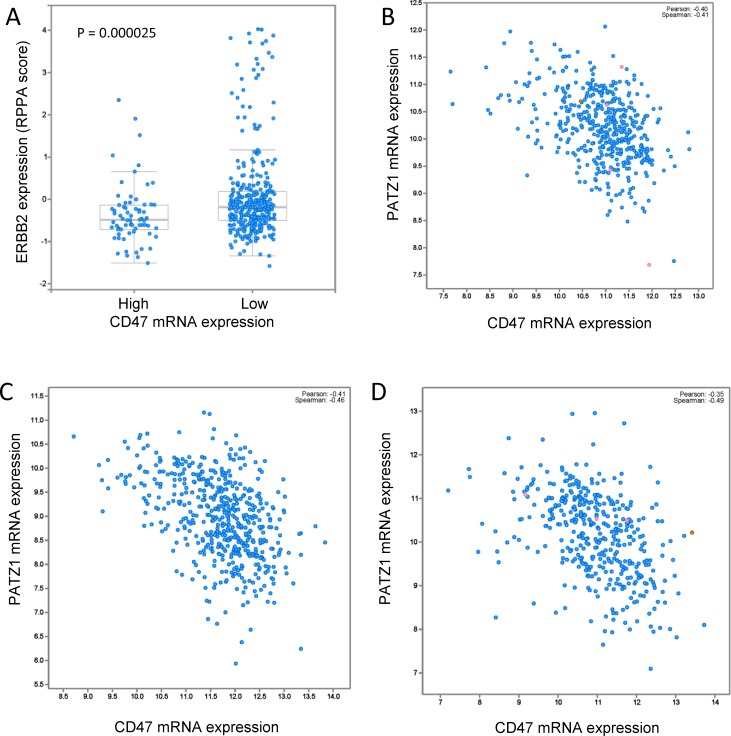
**A.** TCGA breast cancer data for HER2 expression assessed by anti-ERBB2 antibody probing of tumor extracts on a reverse-phase protein array (RPPA). Altered = CD47 mRNA expression elevated >1SD. TCGA data for PATZ1 mRNA expression correlated with CD47 mRNA expression in melanoma **B**., HNSCC **C**., and bladder carcinoma **D**.

The relevance of this *in vitro* bCSC data to human breast cancers was examined using the Cancer Genome Atlas (TCGA) mRNA and protein expression data for invasive breast carcinoma. [37-39]. CD47 mRNA expression >1 SD higher than the mean by RNAseq analysis was significantly associated with decreased overall survival (log rank p-value 0.038, Figure 7A). Because basal breast carcinomas were previously reported to express higher CD47 [9] and have a poorer prognosis, we were concerned that the correlation between survival and CD47 expression may be an artifact of breast cancer heterogeneity. Consistent with the diminished sensitivity of ER^+^ breast cancer cells to B6H12 observed *in vitro*, CD47 mRNA expression in the TCGA dataset was negatively correlated with ER and with HER2 protein expression (*p* = 1.7×10^−6^ and 2.5×10^−5^, respectively, Figure 7B, Figure 8A). Furthermore, CD47 expression was significantly higher in TNBC than in other breast cancers (Figure 7C, *p* = 1.7×10^−9^). Therefore, we excluded other forms of breast cancer and reexamined the correlation between CD47 expression and survival in TNBC (Figure 7D). CD47 expression >1 SD higher than the mean was not associated with decreased survival in these patients (*p* = 0.206).

In contrast to HER2 but consistent with our *in vitro* data, EGFR protein expression in breast tumors positively correlated with CD47 mRNA expression (*p*= 0.009, Figure 7E). A comparison of gene expression altered by B6H12 in bCSC *in vitro* with gene expression significantly correlated with CD47 mRNA expression in the TCGA TNBC primary tumor data was used to identify additional potential targets of CD47 signaling in TNBC that could be regulated by B6H12 (Table 1). Sixty genes achieved significance in both bCSC and TNBC tumor data. Consistent with the protein expression data, EGFR mRNA expression positively correlated with CD47 mRNA expression in TNBC primary tumors (*p* = 0.0007). Five additional genes identified by this analysis (NFAT5, API5, CCDC88A/girdin, EEA1, and IQGAP1) are involved in the EGFR pathway [40-45]. Rab12 regulates autophagy, which mediates the cytoprotective response to CD47 blockade [46]. A number of the identified CD47-dependent genes including MBP1, TBL1XR1, girdin, cyclin D1, CDC27, SPAG9, and JAK1 have reported functions in breast cancer progression [47-54].

**Table 1:** Genes that exhibit significant correlations with CD47 mRNA expression in triple negative primary breast tumors and significant responses to B6H12 treatment in bCSC. Pearson correlation coefficients are presented for the TCGA data

	TNBC TCGA data		TNBC CSC response to B6H12	
Gene	Correlation coefficient (r)	*p*-value	fold change B6H12/control IgG	*p*-value
ITCH	0.399	4.49E-05	1.56	0.0267
ANKRD28	0.389	7.47E-05	1.62	0.00139
NCOA3	0.381	0.000114	1.51	0.0104
SMCHD1	0.376	0.000144	1.78	0.0331
STK4	0.376	0.000147	1.61	0.0235
EHBP1	0.373	0.000165	1.88	0.00932
RAB12	0.371	0.00018	1.78	0.0242
ZNF12	0.371	0.000178	1.52	0.00148
KPNA3	0.365	0.000245	1.62	0.0205
NAA15	0.356	0.000368	1.63	0.00233
BBX	0.355	0.000373	1.57	0.000927
EGFR	0.341	0.000712	−1.91	0.00758
ZBTB38	0.336	0.000855	1.51	0.0378
NFAT5	0.329	0.00115	1.75	0.0187
RIF1	0.327	0.00124	2.56	0.0186
MLL	0.312	0.00223	2.26	0.00890
MLLT10	0.311	0.00229	1.63	0.0176
CHD9	0.304	0.00298	1.60	0.00344
SOX6	0.303	0.00309	1.63	0.0331
ZFP91	0.303	0.00312	1.74	0.00241
SLC25A36	0.300	0.00352	1.52	0.00463
SERBP1	0.299	0.0036	1.58	0.00445
JAK1	0.298	0.0038	1.75	0.0405
NUP153	0.293	0.00449	1.50	0.00537
SLC16A7	0.293	0.00441	1.59	0.00051
C14orf118	0.284	0.006	1.51	6.62E-05
API5	0.282	0.00655	1.52	0.00748
TBL1XR1	0.279	0.00725	1.72	0.00299
EEA1	0.277	0.00769	1.88	0.00820
ANKRD12	0.274	0.00838	1.87	0.00786
SPAG9	0.271	0.00946	1.94	0.00362
UBE2W	0.271	0.00946	1.53	0.0215
SON	0.269	0.00999	1.75	0.00303
BRD4	0.268	0.0102	1.54	0.00749
KIAA1033	0.268	0.0103	1.83	0.0166
IQGAP1	0.260	0.0133	2.69	0.0118
SNTB2	0.246	0.0201	1.52	0.000695
CDC14B	0.245	0.0203	1.70	0.00143
DYRK2	0.243	0.0217	1.54	0.0136
PSME4	0.242	0.0222	1.53	0.0146
FAM126B	0.24	0.0238	1.53	0.00140
GAS2L3	0.24	0.0237	1.78	0.0257
NR1D2	0.24	0.0238	1.73	0.000303
INO80D	0.239	0.024	1.56	0.00170
SPIN1	0.237	0.0256	1.72	0.00939
CDC27	0.234	0.0278	1.78	0.0391
OSBPL8	0.234	0.0282	2.26	0.0162
KTN1	0.231	0.0302	1.51	0.00537
CCDC88A	0.225	0.0353	2.08	0.00365
HECTD1	0.225	0.036	2.04	0.000788
JMJD1C	0.221	0.0398	1.60	0.00522
MBP	0.215	0.0464	1.56	0.0151
EXT1	0.214	0.0468	1.80	0.0112
SMC3	0.214	0.0472	1.63	0.00173
SPEN	0.213	0.0481	1.65	0.0172
CCND1	−0.218	0.0429	1.83	0.0115
PRPF6	−0.246	0.0201	1.74	0.00562
TAF15	−0.252	0.0169	1.61	0.0241
MUM1	−0.321	0.0016	1.55	0.0351
DNAJC1	−0.444	3.53E-06	1.67	0.00439

Several known stem cell markers correlated with CD47 mRNA expression in the TCGA breast carcinoma dataset including a positive correlation with cKit protein expression (Figure 7F, *p* = 0.042). In contrast, phosphorylation of PDK1 at Ser^241^, which induces a cancer stem cell gene expression signature [55, 56], was negatively correlated with CD47 mRNA expression and was the fourth most significant change in protein phosphorylation in the breast cancer dataset (Figure 7G, *p* = 0.0015). The transcription factor PATZ1 maintains stem cells by its regulation of Pou5f1, Nanog, cMyc, and global changes in histone modification [57, 58]. PATZ1 mRNA expression in breast cancers negatively correlated with CD47 mRNA expression (Spearman correlation = −0.41, Figure 7H). Similar negative correlations between PATZ1 and CD47 mRNA expression were found in TCGA datasets for melanoma (−0.41), head and neck squamous cell carcinoma (−0.46), and bladder carcinoma (−0.49) (Figure 8B-8D). Therefore, elevated CD47 expression correlates with known markers and regulators of stem cell maintenance in breast and other cancers.

## DISCUSSION

The premise for developing therapeutic antibodies that target CD47 was that high expression of this cell surface protein protects tumor cells from host innate immune surveillance [16]. However, we previously reported that expression of CD47 in non-transformed cells plays a critical role in regulating stem cell homeostasis. Specifically, CD47 signaling inhibits the expression of cMyc, SOX2, OCT3/4 and KLF4 [20]. Decreasing CD47 in non-transformed cells increases their self-renewal, asymmetric division and ability to reprogram into other differentiated cell types. Because, high CD47 expression limits the stem cell character of non-transformed cells, the high expression of CD47 on cancer stem cells appeared paradoxical, suggesting that the CD47 expressed on CSC may lack the signaling activity to control stem cell self-renewal, either due to alterations in the CD47 or inactivation of pathways that mediate its signaling in CSC. Recent studies in hepatocellular carcinoma stem cells and a breast carcinoma cell line demonstrated that reducing CD47 expression results in loss of stem cell character [17, 19], suggesting that CD47 signaling differentially regulates normal versus malignant stem cells. Conversely, treatment with the CD47 ligand thrombospondin-1 was recently reported to inhibit proliferation, sphere formation, and expression of stem cell transcription factors in Lewis lung carcinoma cells, and CD47 shRNA knockdown blocked this activity [59]. Our results indicate that bCSC express both high levels of CD47 and characteristic stem cell genes. Treatment with B6H12 down regulates KLF4 and inhibits asymmetric cell division of bCSCs, indicating that CD47 is capable of transducing a signal in bCSC. The treated cells resemble differentiated MDA-MB-231 cells and have limited proliferative capacity. This suggests that CD47 expression supports bCSC maintenance.

From a therapeutic perspective, our data indicates that the CD47 antibody B6H12, which was selected for preclinical studies based on its ability to block SIRPα binding [60, 61], has a second activity that could provide therapeutic benefit by suppressing stem cell character in bCSC. B6H12 down regulates EGFR expression at the mRNA and protein levels and inhibits Tyr^1068^ phosphorylation of EGFR. Currently EGFR inhibitors are in phase II clinical trials and show efficacy to inhibit tumor growth in xenograft models [62-65]. The correlation between CD47 and EGFR expression in human breast tumors suggests that therapeutic CD47 antibodies may be effective against tumors with high EGFR expression when used alone or in combination with EGFR inhibitors.

MiR-7 has characteristics of a tumor suppressor in breast cancers and is an emerging cancer therapeutic target [66, 67]. MiR-7 down-regulates expression of KLF4 in breast cancer stem cells [14] and suppresses EGFR mRNA in several cancers by binding to sites in its 3′-UTR [35]. KLF4-dependent suppression of stem cells by miR-7 expression has also been reported in prostate cancer [68]. Thus, B6H12 induction of miR-7-5P could account for the observed activity of B6H12 to decrease EGFR and KLF4 expression in bCSC. Because elevated miR-7 expression was reported to inhibit proliferation and induce apoptosis of breast cancer cells [69], the induction of miR-7 in bCSC by B6H12 may also contribute to the observed inhibition of bCSC proliferation by this CD47 antibody.

B6H12 may also limit tumor growth by inhibiting asymmetric division of CSCs. This activity was observed in a TNBC line, but not in the less aggressive MCF7 cell line or MCF10 immortalized mammary epithelial cells, which showed increased proliferation that is consistent with our primary endothelial cell data [20]. Our data and recent reports that shRNA knockdown of CD47 suppressed CSCs in hepatocellular carcinoma [17] and mammospheres formation in the SUM159 breast carcinoma cell line [19] suggest that direct cell-autonomous effects of therapeutic CD47 antibodies to suppress CSC may extend to additional cancers.

Others have shown that certain CD47 antibodies, but not B6H12, directly induce apoptosis of B-cell chronic lymphocytic leukemia associated with cell shrinkage, decreased mitochondrial transmembrane potential, and phosphatidylserine externalization, but independent of apoptotic caspase activation [70]. These studies suggest that each CD47 antibody may have different effects on CD47 signaling, which may involve direct agonist activities of a given antibody as well as antagonism of the signals induced by SIRPα or TSP1 binding.

In summary, these data demonstrate that CD47 is an active signaling receptor in triple negative human breast carcinoma cells. The CD47 antibody B6H12 directly inhibits cell growth and CSC maintenance in an aggressive subset of the breast cancer cell lines we have tested, whereas stimulation of proliferation was also observed in cell lines with less malignant potential, which is consistent with known CD47 signaling in non-transformed cells. Our EGFR phosphorylation data reveals a novel lateral signaling mechanism through which B6H12 inhibits proliferation of aggressive cancer cells.

## MATERIALS AND METHODS

### Cell culture and reagents

The breast carcinoma cell lines MDA-MB-231, MDA-MB-468, MCF7, MCF10A and T47D1 cells were purchased from ATCC (Manassas, VA) and cultured at 37°C in 5% CO2 using Gibco RPMI 1640 medium with 10% FBS, penicillin, streptomycin, and glutamine (Life Technologies, Grand Island, NY). The bCSCs were cultured using cancer stem cell media from (ProMab, Richmond, CA). APC-conjugated antibodies for EGF and human KLF4 were obtained from R&D Systems. Antibodies specific for EGFR and phospho-Tyr1175 EGFR, actin, NANOG, OCT4, and SOX2 were obtained from Cell Signaling (Danvers, MA), CD47 antibody B6H12 from (Abcam, Cambridge, MA) and Human anti-human CD47-FITC (BD Biosciences). Functional grade purified anti-human CD47-B6H12 and isotype-matched control ant8ibody were from eBioscience, (San Diego, CA), Anti-BrdU APC, EGFR-PE, CD44-FITC, CD24-PE conjugated and Isotype isotype control antibodies were obtained from Bio Legend. Cytochalasin D and anti-tubulin were purchased from Sigma Aldrich. For functional studies B6H12 and its isotype-matched control antibody were used at 1 μg/ml for all experiments throughout the manuscript using RPMI medium containing 2% FBS.

### Asymmetric cell division

MDA-MB-231 cells (ATCC) were labeled with 5-bromo-2′-deoxyuridine (BrdU) for two weeks. The cells were then grown in BrdU-free medium for at least two consecutive cell divisions. The numbers of asymmetric cells were quantified as described [14, 15]. With gentle agitation of the flask, loosely bound bCSCs were separated from adherent MDA-MB-231 cells. bCSCs form loose aggregates after incubation at 37°C. Adherent MDA-MB-231 and enriched bCSCs cells were labeled with BrdU for 10 days and then chased in BrdU free medium for 3-4 days and followed by 2 μM cytochalasin. The cells were immunostained using BrdU antibody and mounted with Vectashield DAPI. The confocal images were taken using a Zeiss 780 microscope at 63X and the asymmetric cell division ratio between cells negative for BrdU and positive for DAPI was calculated [20] [23]. [71]

The MDA-MB-231 cells were labeled with BrdU for two weeks. The cells were grown in BrdU free media at least to two consecutive cell divisions. BrdU free media at 0h and then split into two plates and treated with B6H12 or Isotype control antibody (1 μg/ml) for 5 days. BrdU staining was performed using BrdU Cell Proliferation Kit 2752 from EMD Millipore. BrdU negative and positive for DAPI for DNA segregation was counted manually. The total numbers of DAPI were divided by BrdU negative cells. The untreated or control asymmetric cells were normalized to 1. The ratio of B6H12 was determined as compared to 1 (control).

### Flow cytometry

MDA-MB-231 and bCSCs cells were stained with either isotype control antibody or anti-CD44-FITC and anti-CD24-PE antibodies for 30 min. at room temperature. For intracellular staining, bCSCs cells were stimulated with Leukocyte Activation Cocktail, with GolgiPlusTM (BD Pharmingen) for 6 hours at 37oC before being stained with anti-KLF4 using Foxp3 staining buffer set kit (eBiosciences). Cells were analyzed using a LSR II System flow cytometer (BD Biosciences), and the data were subsequently analyzed and presented using FlowJO software (TreeStar).

### RNA extraction and real-time PCR

CSC-depleted MDA-MB-231 and bCSCs were plated at 1×106 cells in 6-well plates and were-treated with B6H12 or Isotype control antibody (1 μg/ml) for 36 h. Total RNA was extracted using TriPure (Roche). One μg of total RNA was used for first strand cDNA synthesis using a Maxima kit (2-Step RT PCR, Thermo Scientific) according to the manufacturer's instructions. Real time PCR was performed using SYBR Green (Roche) on an MJ Research Opticon I instrument (Bio-Rad) with the amplification program as described in [72]. β2-microglobulin (B2M), HPRT1 or 18S rRNA primers were used as control to normalize mRNA expression.

### Real -time PCR for miRNA-7 expression

MiRNA was extracted using miRNA easy kit from Qiagen as described above. miRNA was subjected to A poly(A) tail reaction and first strand cDNA was amplified using qScript™ microRNA Quantification System according to manufacturer's instructions. miR-7 and SNORD47 primers were purchased from Quanta Bioscience. SNORD47 was used for normalization on Bio-Rad CFX96 Optics Module Instruments.

### BrdU cell proliferation assay

Approximately 8,000 cells were plated per well on 96-well plates and incubated overnight at 37°C. The cells were treated with B6H12 or isotype control antibody (1 μg/ml) for 24 h. BrdU was added for 4 or 24 h as indicated in the figures, and BrdU incorporation was quantified using a BrdU Cell Proliferation Kit according to the manufacturer's instructions (EMD Millipore). For flow analysis, MDA-MB-231 cells were labeled with BrdU, and unlabeled cells were used as a negative control.

### MTT and PKH67 cell proliferation assay

Cell proliferation of differentiated and bCSCs for 0-3 days were analyzed using PKH67 Green Fluorescent Cell Linker Kit according to the manufacturer's conditions via flow cytometry analysis. The raw data of one representative flow cytometric analysis of cell proliferation of differentiated cells (left panel) and bCSCs (right panel are presented in Figure S2H. For relative MFI of three experiments, differentiated and bCSCs were normalized as 100% at day 0.

The bCSCs were treated with B6H12 or isotype control antibody (1 μg/ml) for 3-5 days and were analyzed using the CellTiter 96^®^ Non-Radioactive Cell Proliferation Assay (MTT) Assay according to the manufacturer's instructions.

### Microarray processing and analysis

Samples were prepared according to Affymetrix protocols (Affymetrix, Santa Clara, CA). RNA quality and quantity was ensured using the Bioanalyzer (Agilent, Santa Clara, CA) and NanoDrop (Thermo Scientific, Waltham, MA) respectively. Per RNA labeling, 500 nanograms of total RNA was used in conjunction with the Affymetrix recommended protocol for the HG_U133_Plus 2.0 chips. The hybridization cocktail containing the fragmented and labeled cDNAs was hybridized to the Affymetrix Human HG_U133_Plus 2.0 GeneChip. The chips were washed and stained by the Affymetrix Fluidics Station using the standard format and protocols as described by Affymetrix and the Affymetrix Gene Chip Scanner 3000 was used to scan the probe arrays. Gene expression intensities were extracted using Affymetrix AGCC software. Partek Genomic Suite was used to RMA normalize (Robust Multichip Analysis), summarize, log2 transform the data and run the ANOVA analysis. The raw data is deposited in NCBI Gene Expression Omnibus (GEO): GSE67966

### Mammosphere formation

Cultured MDA-MB-231 cells were washed with IXPBS and Suspension cells (bCSCs) were harvested with gentle agitation from the flask. Mammosphere formation from bCSCs was assessed using cancer stem cell medium from ProMab (Figure 1C) in SmartDish™ (Stemcell Technologies).

To analyze gene set enrichment within our data, we used GSEA reference [73] [74]. The GSEA algorithm computes a ranked list of all genes from a microarray comparison between two conditions and identifies whether individual members of an a priori functionally defined gene set (black vertical bars) are enriched at either the top (red area) or bottom of the ranked genes (blue area) or randomly distributed across the whole ranked gene list, using a modified Kolmogorov-Smirnov statistic. These predefined gene sets are part of a functionally well-established and /or published pathway from databases like KEGG, BioCarta, Reactome and gene ontology. An enrichment score (Green Graph) is calculated based on the level to which a gene set is overrepresented at the top (positive correlation) or bottom (negative correlation) of the ranked gene list and is calculated as the maximum deviation from zero. Genes occurring at the very extreme (dark red or dark blue area) on either side of the ranked list are weighted more heavily compared with genes occurring in the middle (light red or light blue area) of the ranked gene list that contain genes that are not differentially expressed. Statistical significance is defined by the p-value, which is also adjusted for multiple hypothesis testing. A gene set-based permutation test of 1.000 permutations was applied and genes were ranked according to Student's t statistic. All other parameters were set to GSEA defaults. The Broad Molecular Signatures Database v5.0 (MSigDB), actually consist of over 4000 different gene sets. Alternatively, we used microarray datasets from the GEO database to derive gene sets that we then used for GSEA analysis [5, 6]. http://www.broadinstitute.org/gsea/. http://www.broadinstitute.org/gsea/. The analyzed listed genes were attached as excel sheet Attached Vs stemness.

### Immunoprecipitation and western blots

bCSC-depleted MDA-MB-231 cells and bCSCs were plated at 1×106 cells/well in 6-well plates. The cells were serum-starved for 2 hours using serum-free RPMI medium. The cells were pre-treated with B6H12 (1 μg/ml) for 20 min. Cell lysates were made using immunoprecipitation buffer (50 mM Tris-HCl, 150 mM NaCl, and 1% Nonidet P-40) along with 1× Complete Mini-protease inhibitor mixture (Roche Applied Science). Cell lysates were centrifuged at 13,000 rpm for 15 min. A BCA assay (Thermo Scientific) was used to quantify total protein. Dynabeads (Invitrogen) were used for Immunoprecipitation. The Dynabeads were washed three times with activation buffer. The cell lysates were incubated in Dynabeads-protein G along with anti-EGFR and CD47 antibodies (1:500) and incubated for 24 hours at 4°C on a shaker. The beads were washed three times with lysate buffer and heated at 95°C for 5 min. The immunoprecipitated cell lysates were loaded on 4-12% NuPAGE gels (Life Technologies), and Western blotting was performed. For immunoprecipitation, primary antibody against phospho-EGFR (1:1000) was used. Normalization of protein lysates used for Western blotting was performed by reprobing with anti-β-actin (1:3000) or tubulin or EGFR antibodies. The western blot intensity was measured using Image J program.

### CD47 and EGFR immunoprecipitation

MDA-MB-231 cells were pretreated with B6H12 (1 μg/ml) for 15 min. The cells were further treated with EGF for 7 min and total Lysate were performed using NP-40 lysis buffer as described above. CD47 and EGFR immunoprecipitation was performed using Dynabeads® Protein G Immunoprecipitation Kit (Life technologies) according to manufacturer's instructions with slight modifications in incubation time (3 h) for EGFR and CD47 antibodies with Dyna beads. The immunoprecipitated cell lysates were loaded on 4–12% NuPAGE gels (Life Technologies) and transferred using iBlot® - Western Blotting System (Life Technologies), The membrane was blocked with 3% milk with addition of Complete mini Pellet for 20 minutes. IP-western blots were performed using -EGFRY1068, EGFR and CD47 antibodies (1:1000) overnight at 4°C. The membrane was washed two times with TBST for 10 minutes. Secondary HRP (Amersham), IRDye 800 or 680 (LI-COR) 1:3000 were used for 1h at RT. The membranes were further washed 3 times for 10 minutes interval. The images were captured by using WesternSure PREMIUM Chemiluminescent Substrate with Odyssey® Fc(LI-COR). The membranes were immunoblotted with EGFR and CD47 for total IP-input.

### Immunostaining and confocal microscopy

bCSCs and differentiated cells were plated on Lab Tek 8-well chamber slides using 2% FBS. The cells were pre-treated with B6H12 (1 μg/ml) for 15 min and stimulated with EGF (30 ng/ml) for 10 min. The cells were fixed using paraformaldehyde for 15 min and rapidly washed 2 times with 1X PBS. The cells were permeabilized using 0.14% Triton X-100 and 3% BSA in PBS-Tween for 5 min and washed three times for 5 min each. Anti-KLF44 antibody was diluted 1:250 using 1% BSA in PBS-Tween and incubated for overnight at 4°C on a shaker. The cells were washed twice with 1XPBS for 5 min each. 1:500 anti-mouse-Alexa488 (Life Technologies) were used to detect binding of the primary antibodies. The cells were washed three times with 1xPBS and mounted using DAPI VECTASHIELD® Mounting Medium (Vector Laboratories, Inc, Burlingame, CA). The images were acquired using Zeiss 710 or Zeiss 780 microscopes at 63X.

### TCGA invasive breast carcinoma patient tumor data

mRNA (RNA Seq V2 RSEM) and protein expression data from the TCGA Breast Invasive Carcinoma raw data at the NCI was analyzed using cBioPortal tools [38] [39]. The TCGA breast invasive carcinoma (BRCA) Level 3 RNAseq gene expression data and clinical information were downloaded from https://genome-cancer.ucsc.edu/proj/site/hgHeatmap/ (file: TCGA_BRCA_exp_HiSeqV2-2015-02-24.tgz). The data from 1095 primary tumors was used in mRNA expression analysis and survival analysis. For survival analysis, log-rank tests were done using the survival R package. Expression values of CD47 were dichotomized into high and low expression using mean + 1SD as a cutoff.

### Statistical analysis

The p-values for asymmetric cell division, cell proliferation, cell/western blot imaging intensity and flow MFI were measured using the t-test for two samples assuming equal variances. The p-value less than ≤ 0.05 were used as statistically significant. *, ** and *** corresponding to ≤ 0.05, 0.05 and 0.005 respectively. The real–time PCR was measured using either t-test or ANOVA: Two-Factor with replication, a p-value ≤0.05 was considered significant.

## SUPPLEMENTARY MATERIAL FIGURES AND TABLES














