# Supplementary material for: A function-blocking CD47 antibody suppresses stem cell and EGF signaling in triple-negative breast cancer
Source: Oncotarget. 2016 Jan 31;7(9):10133–52. doi: 10.18632/oncotarget.7100 (PMC4891109; doi:10.18632/oncotarget.7100)
Supplement: Supplementary file 1 [file oncotarget-07-10133-s001.pdf]

A function-blocking CD47 antibody suppresses stem cell and EGF signaling in triple-negative breast cancer

Supplementary Material

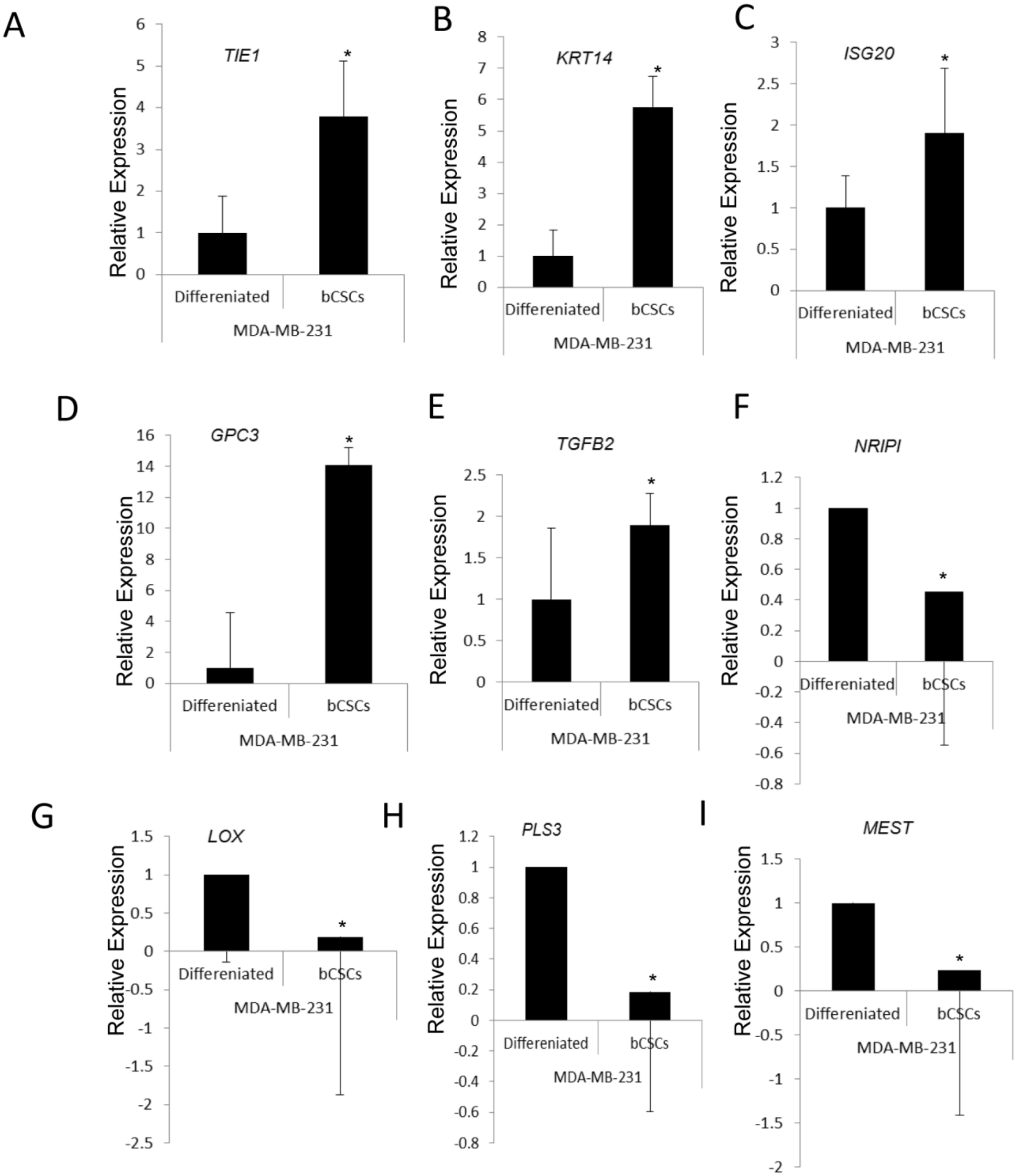

Fig. S1.* *(A-I) Relative expression of gene enrichment for stemness markers TIE1, KR14, ISG20, GPC3, TGFB2, NRIPI, LOX, PLS3 and MEST in Differentiated and bCSCs isolated form the MDA-MB-231 cell line. 18S is used as internal control for real time analysis * represent significant (P-0.05) using t-test

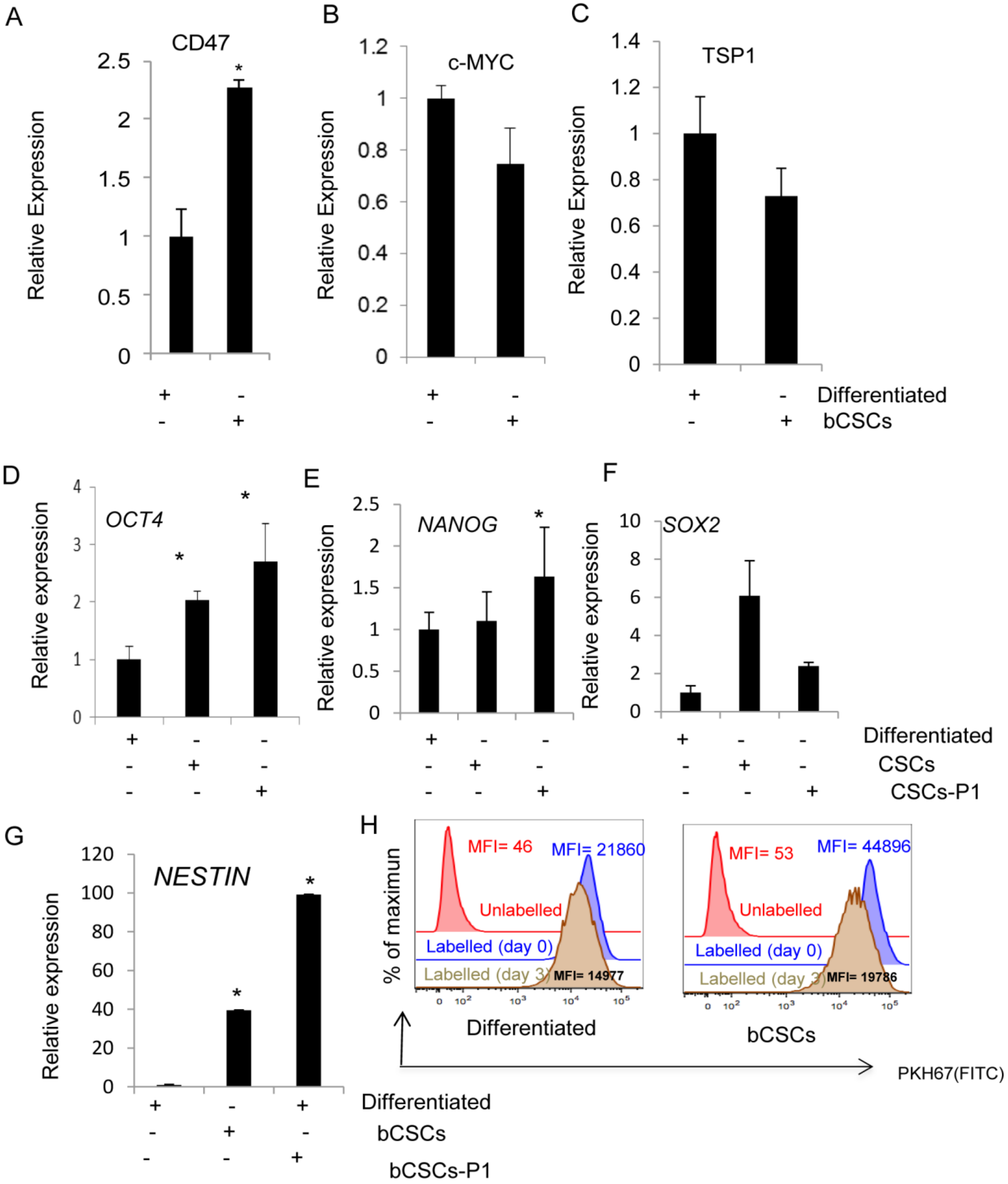

Fig. S2. (A-G) Relative expression of CD47, c-MYC and TSP1 in Differentiated and bCSCs isolated form the MDA-MB-231 cell line. Actin is used as control for real time PCR analysis. (D-G) Relative Expression of OCT4, NANOG, SOX2 and NESTIN in Differentiated and bCSCs. B2M is used as internal control for real time PCR analysis. Anova: Two-Factor with Replication P value ≤0.05 (*). (H) Raw data of Flow cytometric analysis of cell proliferation of differentiated cells (left panel) and bCSCs (right panel).

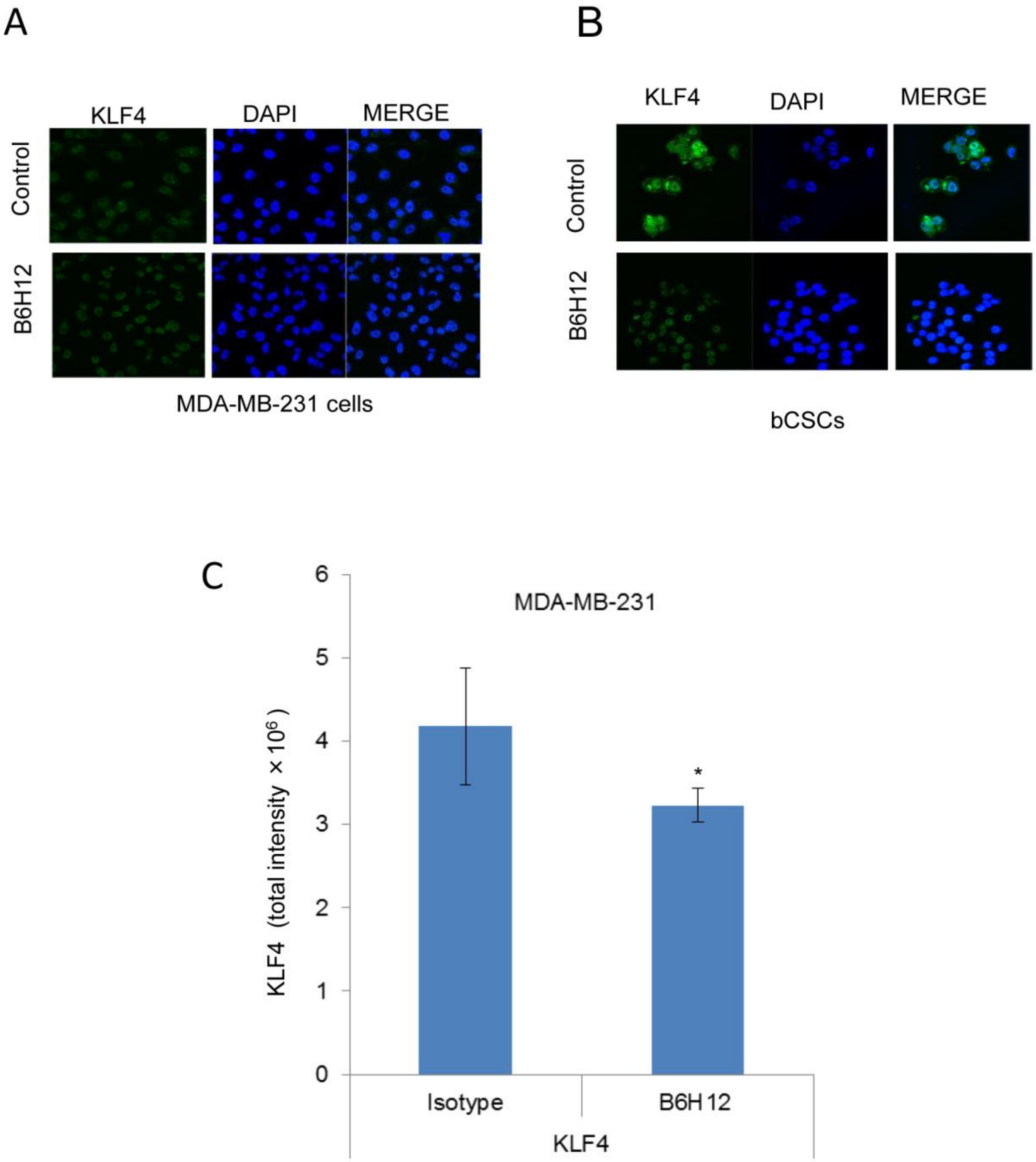

Fig. S3. B6H12 treatment of bCSC reduces expression of KLF4. (A and B). MDA-MB-231 cells and bCSCs were plated on labTek 8 well chamber slides using medium containing 2% FBS for 24 h at 37°C. The cells were pre-treated with B6H12 for 24 h. The cells were immunostained with KLF4 antibody, and images were captured using a Zeiss 710 confocal microscope. (C) Total intensity of KLF4 positive cells were calculated and t-test was performed.

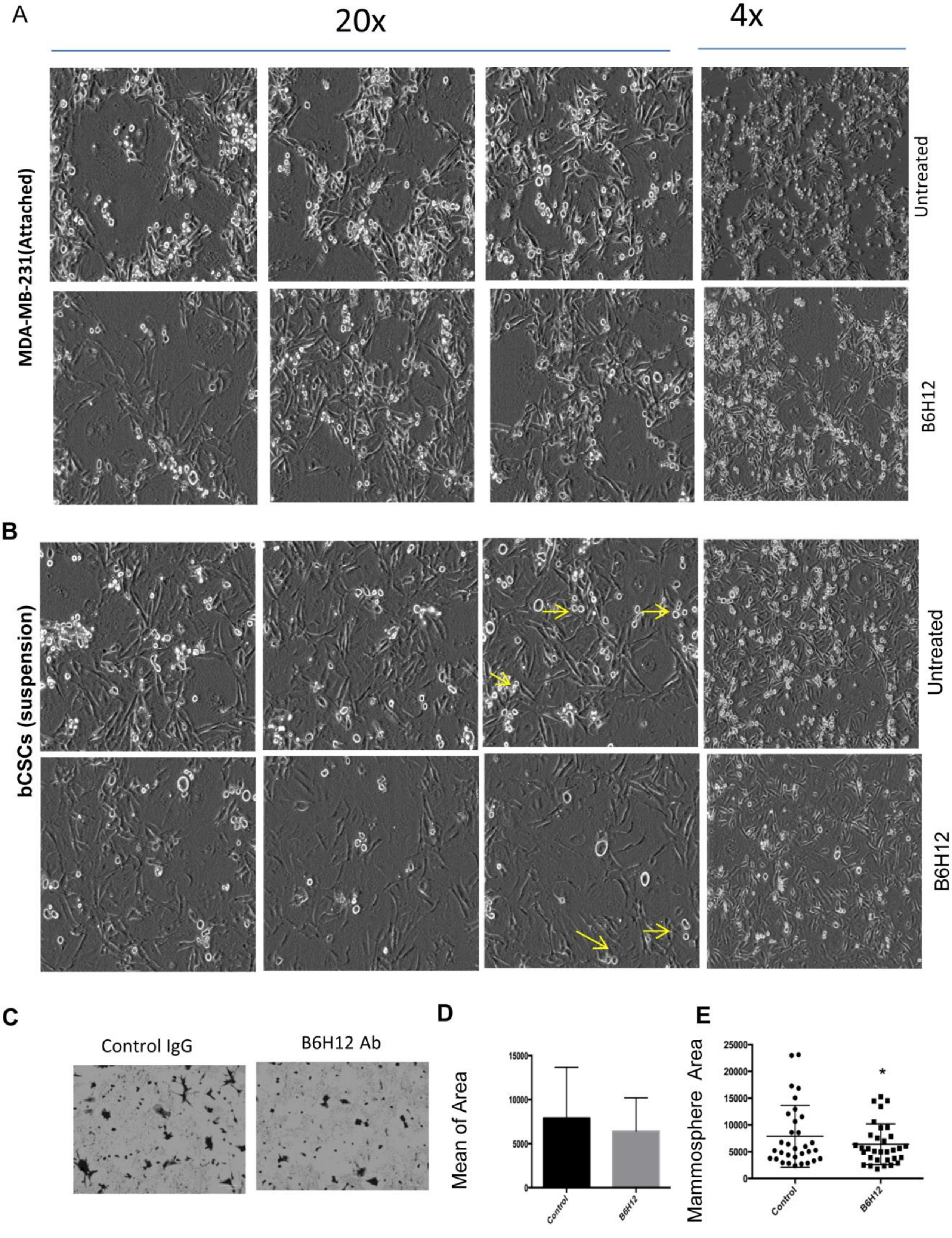

FIG. S4. **CD47 blocking antibody (B6H12) induces morphological change and reduce number of aggregates only in bCSCs but not in CSC-depleted MDA-MB-231 cells.** (A & B) MDA-MB-231 and bCSCs were treated in the presence or absence of 1µg/ml of B6H12 for 72 h. The images of live cells were captured using an Olympus microscope at 20X and 4X. **(C).** bCSCs were treated with control or 1µg/ml of B6H12 for 10 days using cancer stem cell media form aggregates. **(D&E)** quantification of mamosphere size. t-test P value ≤0.001 (*).

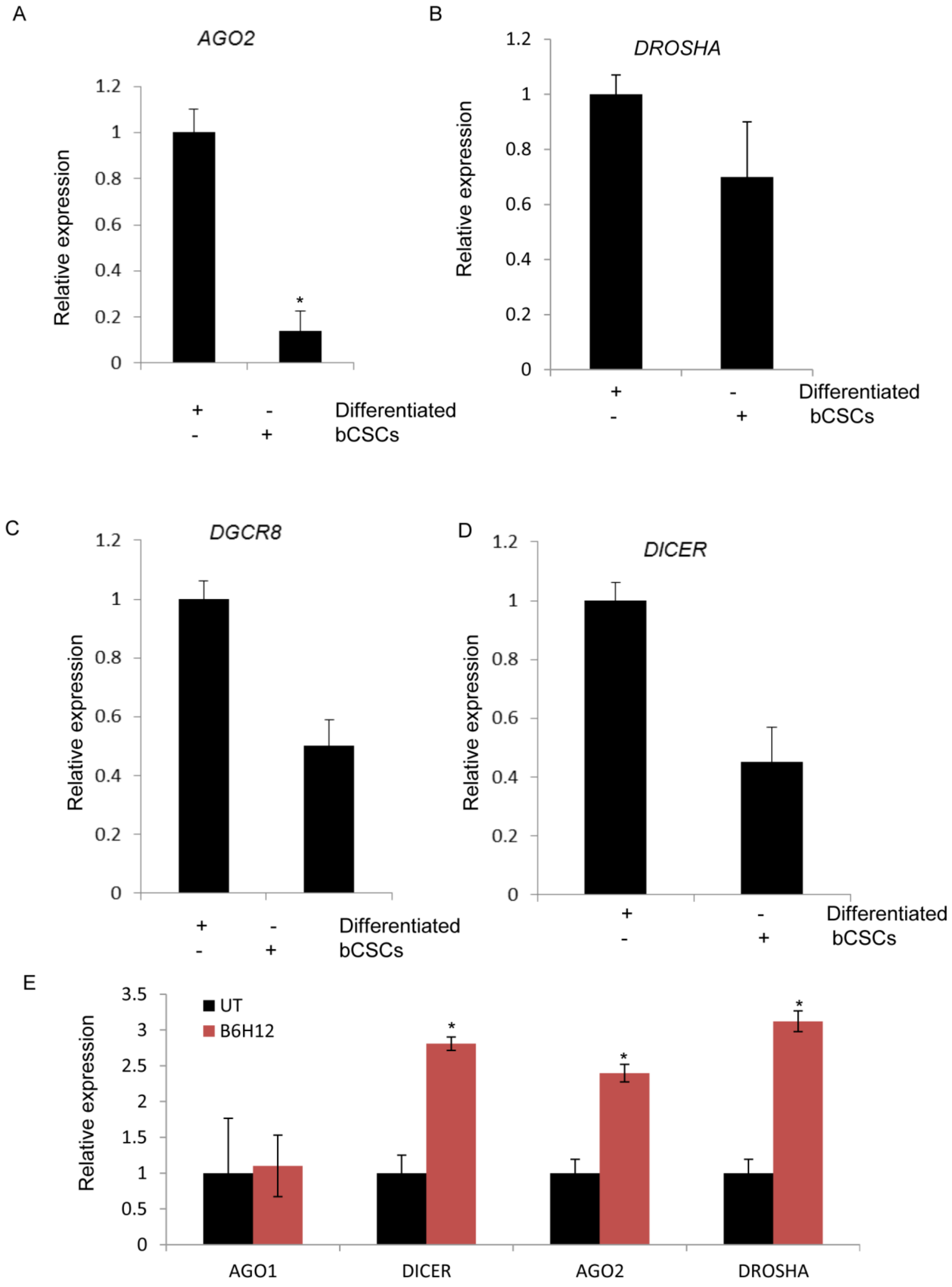

Fig. S5. (A-D) ***Relative expression of*** RNA-induced silencing complex between differentiated and bCSCs derived from MDA-MB-231 cells.(E) B6H12 treatment increase RNA-induced silencing complex. Anova: Two-Factor with Relpcation P value ≤0.05 (*).

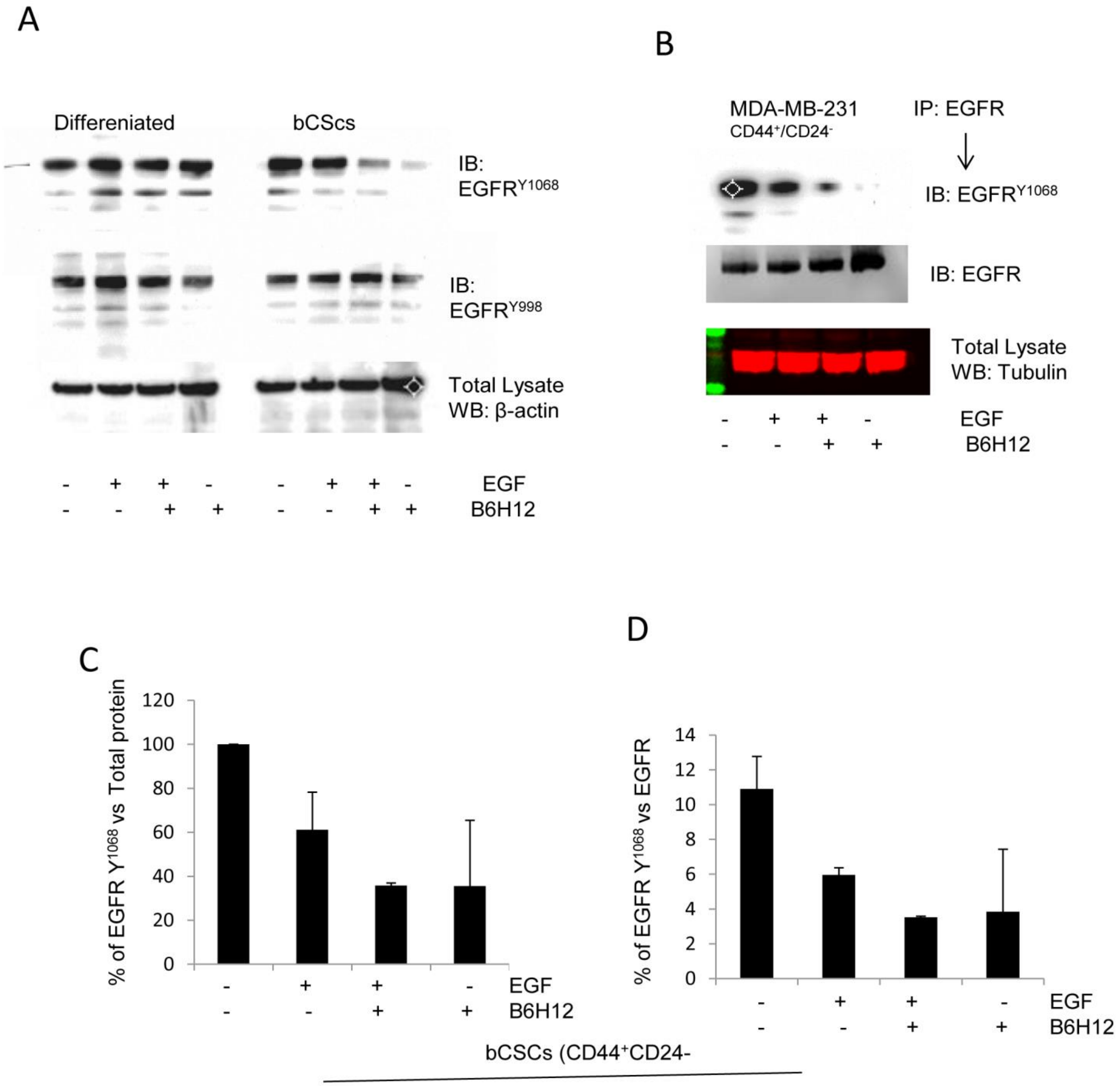

Fig. S6. (A) B6H12 does not inhibit EGHFRY992 B6H12 . (B) IP-western blot of EGFR was performed using CD44high and CD24 low sorted cells under similar conditions as above (C and D) Quantification of EGFRY1068 using Image J program with respect to total tubulin and EGFR control. (n=2))
