# Supplementary material for: A function-blocking CD47 antibody suppresses stem cell and EGF signaling in triple-negative breast cancer
Source: Oncotarget. 2016 Jan 31;7(9):10133–52. doi: 10.18632/oncotarget.7100 (PMC4891109; doi:10.18632/oncotarget.7100)
Supplement: Supplementary file 2 [file oncotarget-07-10133-s002.pdf]

Supplementary Table 1:

Total gene =98

bCSCs vs Differentiated (up 8)

Gene Symbol	Gene Title	Fold-Change (Suspended vs. Attached)
AKR1C3	aldo-keto reductase family 1, member C3 (3-alpha hydroxysteroid dehydrogenase, type II)	1.81864
AKR1C2	aldo-keto reductase family 1, member C2 (dihydrodiol dehydrogenase 2; bile acid binding	1.67318
AKR1C1	aldo-keto reductase family 1, member C1 (dihydrodiol dehydrogenase 1; 20-alpha (3-alpha	1.65919
TRIM16	tripartite motif containing 16	1.51663
HLA-DRA	major histocompatibility complex, class II, DR alpha	1.51385

bCSCs vs Differentiated (down 90)

Gene Symbol	Gene Title	Fold-Change (MDA231_Suspended vs. MDA231_Attached)
TFF1	trefoil factor 1	-4.20243
ATRX	alpha thalassemia/mental retardation syndrome X-linked	-2.48738
ANKRD36B	ankyrin repeat domain 36B	-2.35466
SORBS2	sorbin and SH3 domain containing 2	-2.2884
TOP1	topoisomerase (DNA) I	-2.259
NCF2	neutrophil cytosolic factor 2	-2.1951
TNFSF10	tumor necrosis factor (ligand) superfamily, member 10	-2.16617
INHBA	inhibin, beta A	-2.10742
CDC42BPA	CDC42 binding protein kinase alpha (DMPK-like)	-2.09505
CCDC88A	coiled-coil domain containing 88A	-2.07101
MALAT1	metastasis associated lung adenocarcinoma transcript 1 (non-protein coding)	-2.05448
SPAG9	sperm associated antigen 9	-2.05225
INHBA	inhibin, beta A	-2.04851
KIAA1199	KIAA1199	-2.04464
RIF1	RAP1 interacting factor homolog (yeast)	-2.01239
CD24	CD24 molecule	-1.88383
