# Supplementary material for: A function-blocking CD47 antibody suppresses stem cell and EGF signaling in triple-negative breast cancer
Source: Oncotarget. 2016 Jan 31;7(9):10133–52. doi: 10.18632/oncotarget.7100 (PMC4891109; doi:10.18632/oncotarget.7100)
Supplement: Supplementary file 5 [file oncotarget-07-10133-s005.pdf]

B6H12 _Ab down vs Ctrl_Ab (1)

Gene Symbol	Gene Title	Fold-change (B6H12_Ab vs. Ctrl_Ab)
EGFR	epidermal growth factor receptor	-1.9058
---	---	-1.55596

B6H12 _Ab up vs Ctrl_Ab (224)

Gene Symbol	Gene Title	Fold-Change (B6H12_Ab vs. Ctrl_Ab)
NIPBL	Nipped-B homolog (Drosophila)	2.92333
ASPM	asp (abnormal spindle) homolog, microcephaly associated (Drosophila)	2.83547
WASL	Wiskott-Aldrich syndrome-like	2.82213
NASP	Nuclear autoantigenic sperm protein (histone-binding)	2.75136
IQGAP1	IQ motif containing GTPase activating protein 1	2.68542
TPR	translocated promoter region, nuclear basket protein	2.62456
TPR	translocated promoter region, nuclear basket protein	2.56223
RIF1	RAP1 interacting factor homolog (yeast)	2.46908
WASF2	WAS protein family, member 2	2.36071
ATRX	alpha thalassemia/mental retardation syndrome X-linked	2.31098
TOP1	topoisomerase (DNA) I	2.13487
DICER1	dicer 1, ribonuclease type III	1.5686
